# Radiosynthesis automation, non-human primate biodistribution and dosimetry of K^+^ channel tracer [^11^C]3MeO4AP

**DOI:** 10.1186/s13550-024-01092-8

**Published:** 2024-04-29

**Authors:** Yu-Peng Zhou, Moses Q. Wilks, Maeva Dhaynaut, Nicolas J. Guehl, Danielle R. Vesper, Sung-Hyun Moon, Peter A. Rice, Georges El Fakhri, Marc D. Normandin, Pedro Brugarolas

**Affiliations:** 1 Department of Radiology, Massachusetts General Hospital and Harvard Medical School, 55 Fruit St, Bulfinch 051, Boston, MA 02114 USA; 2 Present Address: Department of Radiology and Biomedical Imaging, Yale School of Medicine, New Haven, CT 06520 USA

**Keywords:** Voltage-gated potassium channel, [^11^C]3MeO4AP, Automation, cGMP, PET tracer, Biodistribution, Radiation dosimetry

## Abstract

**Background:**

4-Aminopyridine (4AP) is a medication for the symptomatic treatment of multiple sclerosis. Several 4AP-based PET tracers have been developed for imaging demyelination. In preclinical studies, [^11^C]3MeO4AP has shown promise due to its high brain permeability, high metabolic stability, high plasma availability, and high in vivo binding affinity. To prepare for the translation to human studies, we developed a cGMP-compatible automated radiosynthesis protocol and evaluated the whole-body biodistribution and radiation dosimetry of [^11^C]3MeO4AP in non-human primates (NHPs).

**Methods:**

Automated radiosynthesis was carried out using a GE TRACERlab FX-C Pro synthesis module. One male and one female adult rhesus macaques were used in the study. A high-resolution CT from cranial vertex to knee was acquired. PET data were collected using a dynamic acquisition protocol with four bed positions and 13 passes over a total scan time of ~ 150 min. Based on the CT and PET images, volumes of interest (VOIs) were manually drawn for selected organs. Non-decay corrected time-activity curves (TACs) were extracted for each VOI. Radiation dosimetry and effective dose were calculated from the integrated TACs using OLINDA software.

**Results:**

Fully automated radiosynthesis of [^11^C]3MeO4AP was achieved with 7.3 ± 1.2% (n = 4) of non-decay corrected radiochemical yield within 38 min of synthesis and purification time. [^11^C]3MeO4AP distributed quickly throughout the body and into the brain. The organs with highest dose were the kidneys. The average effective dose of [^11^C]3MeO4AP was 4.0 ± 0.6 μSv/MBq. No significant changes in vital signs were observed during the scan.

**Conclusion:**

A cGMP-compatible automated radiosynthesis of [^11^C]3MeO4AP was developed. The whole-body biodistribution and radiation dosimetry of [^11^C]3MeO4AP was successfully evaluated in NHPs. [^11^C]3MeO4AP shows lower average effective dose than [^18^F]3F4AP and similar average effective dose as other carbon-11 tracers.

**Supplementary Information:**

The online version contains supplementary material available at 10.1186/s13550-024-01092-8.

## Introduction

**Scheme 1.**
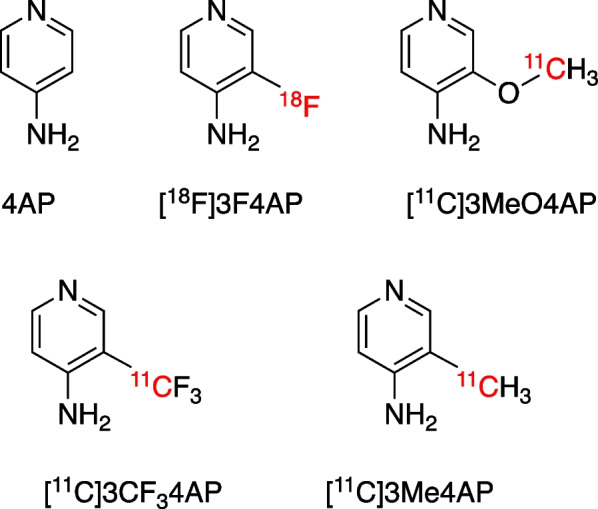
Chemical structures of 4-aminopyridine (4AP) and related PET tracers

4-Aminopyridine (4AP) is a voltage-gated potassium channel blocker (Scheme 1), which binds inside the pore of potassium channels under protonated condition [1, 2]. 4AP has been approved by the U.S. Food and Drug Administration for the symptomatic treatment of multiple sclerosis (MS) [3–5]. Upon demyelination, axonal potassium channels K_v_1.1 and K_v_1.2 normally located under the myelin sheath become exposed and increase in expression. 4AP binds to the potassium channels in demyelinated axons, reducing the abnormal efflux of K^+^ ions and restoring axonal conduction [6, 7]. Several 4AP based PET tracers have been developed by our group (Scheme 1) [8–11]. The fluorine-18 based PET tracer [^18^F]3-fluoro-4-aminopyridine ([^18^F]3F4AP) has been characterized in healthy non-human primates and healthy human subjects, showing selective binding to potassium channels, high brain penetration, high metabolic stability, high plasma availability, high reproducibility, high specificity, and fast kinetics [8]. In addition, [^18^F]3F4AP showed high sensitivity to a traumatic brain injury (TBI) in a non-human primate [12]. Clinical trials of [^18^F]3F4AP in people with multiple sclerosis (NCT04699747), neurodegeneration and traumatic brain injury patients (NCT04710550) are currently underway.

Carbon-11 is one of the most commonly used PET radionuclides. The half-life of carbon-11 (t_1/2_ = 20.3 min) is shorter than that of fluorine-18 (t_1/2_ = 110 min). Due to their shorter half-life, carbon-11 PET tracers generally display lower effective doses and offer the opportunity to scan the same subject twice on the same day [13–18]. Several carbon-11 labeled 4AP-based PET tracers have been reported including 3-[^11^C]trifluoromethyl-4-aminopyridine ([^11^C]3CF_3_4AP) [9, 19], 3-[^11^C]methoxy-4-aminopyridine ([^11^C]3MeO4AP) [10], and 3-[^11^C]methyl-4-aminopyridine ([^11^C]3Me4AP) [11] (Scheme 1). Among them, [^11^C]3MeO4AP was the only one that showed marked uptake in a three-year old focal traumatic brain injury in a rhesus macaque. Additionally, [^11^C]3MeO4AP showed several promising properties such as high brain permeability, high metabolically stability, high plasma availability, and higher in vivo binding affinity and specificity to potassium channels compared with [^18^F]3F4AP [10]. The properties mentioned above make [^11^C]3MeO4AP a promising candidate for imaging demyelinating diseases. In order to bring [^11^C]3MeO4AP to a clinical study, the cGMP (Current Good Manufacturing Practices) production of PET tracer and the radiation dosimetry evaluation are required to fulfill the IND (Investigational New Drug) application requirement. In this paper, we describe the cGMP-compatible automated radiosynthesis and quality control of [^11^C]3MeO4AP and its biodistribution and radiation dosimetry in non-human primates.

## Experimental section

### Non-human primates

One male adult (M1) and one female adult (M2) rhesus macaque were used in this study. Animal body weights on the days of imaging were 13.68 kg (Male) and 9.74 kg (Female). Prior to the imaging session, animals were sedated with ketamine/xylazine (10/0.5 mg/kg IM) and were intubated for maintenance anesthesia with isoflurane (1–2% in 100% O_2_). A venous catheter was placed in the saphenous vein for radiotracer injection and, an arterial catheter was placed in the posterior tibial artery for blood sampling. The animal was positioned on a heating pad on the bed of the scanner for the duration of the study. During the 150 min of PET scan, vital signs including temperature, blood pressure and oxygen saturation, heart rate, respiratory rate, and exhaled CO_2_ were continuously monitored.

### PET tracer production

*Automated radiosynthesis of [*^*11*^*C]CH*_*3*_*I*: The proton bombardment (40 µA, 3–7 min) of nitrogen gas in the presence of oxygen (1%) generated [^11^C]CO_2_ via ^14^N(p,α)^11^C nuclear reaction. The cyclotron generated [^11^C]CO_2_ was transferred into a GE TRACERlab FX MeI module or TRACERlab FX C Pro synthesis module and then mixed with hydrogen. The mixture was passed over nickel at 350 °C to produce [^11^C]CH_4_, which was then circulated and passed through a high-temperature iodine bed. The sublimated iodine was reacted with [^11^C]CH_4_ in gas phase to generate [^11^C]CH_3_I, which was first trapped in MeI trap and then released, transferred into a V-vial (semi-automated method) or to the reactor (fully automated method using TRACERlab FX C Pro synthesis module) for the radiomethylation reactions.

*Semi-automated radiosynthesis of [*^*11*^*C]3MeO4AP*: 3–5 mg of 3-hydroxyl-4-aminopyridine, 300 μL DMSO, and 5 μL of 5N NaOH solution were added into a 5 mL V-vial. The mixture was vortexed for 1 min and nitrogen gas was bubbled through the solution for 3 min until the solution turned pink. The produced [^11^C]CH_3_I was then bubbled into the mixture for 3 min at room temperature. All the needles were removed and the sealed V-vial was heated at 90 °C for 5 min. When reaction was completed, 2.5 mL of H_2_O was added via syringe and the mixture was transferred to the prep-HPLC for purification. The tracer was purified using a semiprep HPLC column using 10 mM sodium phosphate (pH 8) mobile phase containing 5% ethanol (Waters XBridge C18 column, 10 × 250 mm, radio-detector, UV detector = 254 nm, flow rate = 4 mL/min). The HPLC fraction containing the product (approx. 6–8 min) was diluted with 10 mL of 0.9% sodium chloride for injection and filter-sterilized using a 0.22 μm PES filter (Millex-GP, Millipore). The identity and purity of [^11^C]3MeO4AP was confirmed by analytical HPLC with co-injected nonradioactive reference as standard (Thermo Scientific Dionex UltiMate 3000 UHPLC System; Analytical HPLC conditions: 5% MeOH + 95% 10 mM NH_4_HCO_3_, pH = 8, flow rate = 1 mL/min. XBridge C18 column, 3.5 μm, 4.6 × 100 mm, radio-detector, UV detector = 254 nm; 10 μL of sample injected).

**Fig. 1:**
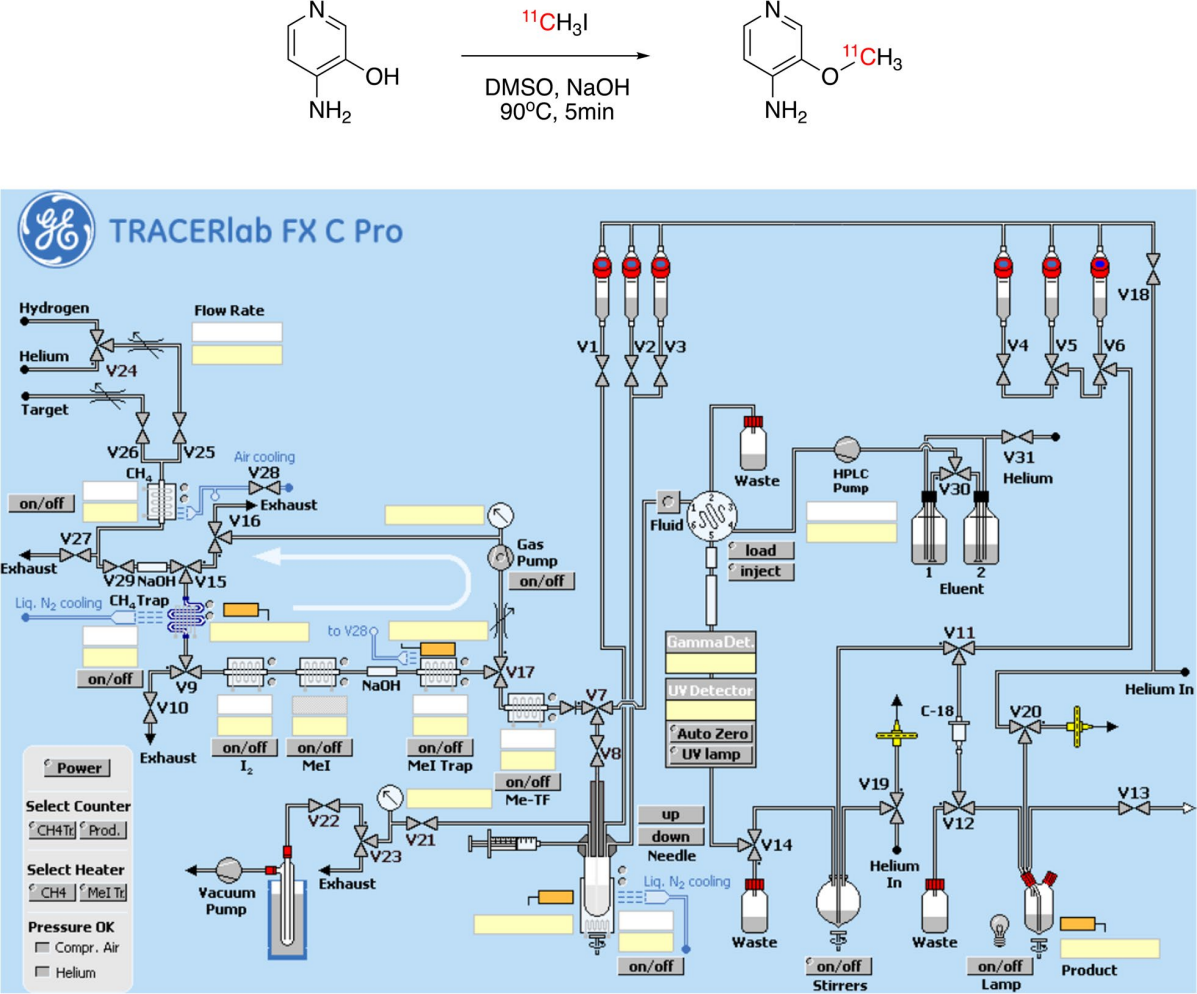
Radiosynthesis of [^11^C]3MeO4AP (top), and control panel of GE TRACERlabT FX C Pro synthesizer (bottom)

*Fully automated radiosynthesis of [*^*11*^*C]3MeO4AP*: The fully automated radiosynthesis was carried out using a GE TRACERlab FX C Pro synthesis module. DMSO solution of 3-hydroxyl-4-aminopyridine containing base was prepared similar to the semi-automated radiosynthesis method. The mixture was preloaded in the reactor of the synthesis module. 1 mL of H_2_O was loaded into vial-3 (Fig. 1). The produced [^11^C]CH_3_I was send to the reactor via valve 8 at room temperature. The mixture was heated at 90 °C for 5 min. 1 mL H_2_O from vial 3 was added to the mixture and the solution was transferred to the prep-HPLC for purification using the same condition. The whole process was preprogrammed and ran automatically. The identity and purity of [^11^C]3MeO4AP was confirmed by the same method used in semi-automated radiosynthesis protocols.

*Quality control (QC) tests*: The following QC tests were carried out. Appearance was checked by visual inspection. Radiochemical identity was confirmed by coelution of nonradioactive 3MeO4AP and [^11^C]3MeO4AP on radioHPLC. Molar activity was calculated by dividing the dose measured in a dose calibrator by the moles calculated using a calibration curve from HPLC. Radiochemical purity was calculated as the ratio of the area under the curve (AUC) of the product peak on radioHPLC to all other radioactive peaks. Radionuclidic identity was confirmed by calculating the half-life from two radioactivity measurements > 10 min apart using a dose calibrator and by gamma spectroscopy. Filter membrane integrity test was performed using the bubble point test. Bacterial endotoxin testing was performed using the Endosafe device from Charles River. Chemical purity was estimated by integrating all the UV active peaks on HPLC at 254 nm. Tracer stability was performed by analytical radio-HPLC on a dose sample 2 h after injection. Finally, the pH was measured using pH test strips.

### PET tracer administration

Radiotracer solution (10 mL) was administered via the lateral saphenous vein over a 3-min infusion. All injections were performed using syringe pumps (Medfusion 3500). After administration of the dose, the catheter was flushed with 10 mL of saline and the residual activity in the syringe and catheter measured to calculate the amount of activity.

### Image acquisition protocol

Imaging was performed on a GE Discovery MI PET/CT scanner. Subjects were positioned on the scanner bed and a CT from the cranial vertex to the knee was acquired. Based on the length of the animals determined from the CT images, 4 bed positions with overlapping edges were selected for PET acquisition (25 cm per bed position with 2.5 or 5 cm overlap on each end). PET images were acquired over a period of 2.5 h. The PET acquisition protocol consisted of a series of static images at each bed position of increasing duration starting upon administration of the tracer. The full acquisition protocol was as follows: high resolution CT, 5 passes × 1 min/bed, 5 passes × 3 min/bed, 3 passes × 5 min/bed. After completion of the scan, the PET data was reconstructed using the scanner’s OSEM with PSF and TOF modeling reconstruction algorithm with 34 subsets and 2 iterations applying the corrections for scatter, attenuation, deadtime, random coincidence and scanner normalization.

### Blood sampling

1 mL arterial blood samples were taken at approximately 3, 5, 10, 15, 30, 60, 90, 120 and 150 min post-injection through an arterial catheter. Blood samples’ radioactivity was counted using a calibrated gamma counter.

### Image analysis and dosimetry calculation

The imaging analysis was carried out using PMOD software. Based on the high resolution CT and PET images, representative subvolumes of organs of interest (VOIs) were manually drawn for the following tissues: adrenals, brain, breasts, gall bladder, small intestine, upper and lower large intestine, stomach, heart contents, heart muscle, kidney, liver, lung, muscle, ovaries, pancreas, red marrow, trabecular and cortical bone, spleen, testes, thymus, thyroid, urinary bladder and uterus. No partial volume correction was applied. Time-activity curves (TACs) were extracted for each VOI. For dosimetry calculation, TACs were uncorrected for decay and extrapolated to ten half-lives after injection by assuming that any further decline in radioactivity occurred only due to physical decay with no biological clearance. OLINDA v1.0 was used to calculate effective dose, using adult male and female phantoms for the male and female primates, respectively. Effective doses were calculated directly from OLINDA using ICRP60 organ weighting factors, and OLINDA output was used to calculate effective doses using updated ICRP103 organ weightings.

## Results

### Automation of radiosynthesis

The semi-automated radiosynthesis of PET tracer [^11^C]3MeO4AP was achieved using a similar method as previously reported [10]. Starting from 10.73 to 16.65 GBq (290 mCi–450 mCi) of [^11^C]CH_3_I, 1.37–1.89 GBq (37 mCi–51 mCi) of [^11^C]3MeO4AP was synthesized in 11.3 ± 2.1% (n = 4) non-decay corrected radiochemical yield and > 99% of radiochemical purity in ~ 45 min of synthesis and purification time. The molar activity at EoS was 51.8 ± 3.7 GBq/µmol (1.4 ± 0.1 Ci/μmol).

In order to fulfill the cGMP requirements, a fully automated radiosynthesis method was developed using GE TRACERlab FX C Pro automatic synthesizer (Fig. 1). The module contains a [^11^C]CH_4_ synthesis module, a needle reactor, a prep-HPLC, and a formulation system. The radiosynthesis of [^11^C]3MeO4AP was preprogrammed and ran automatically. A typical automated radiosynthesis starts from 5.55–6.66 GBq (150–180 mCi) of [^11^C]CH_3_I and yields 0.38–0.46 GBq (10.4–12.47 mCi) of [^11^C]3MeO4AP in 7.3 ± 1.2% (n = 4) of non-decay corrected radiochemical yield and 99% of radiochemical purity in 38 min of synthesis and purification time. Analytical HPLC chromatograms of the product and coinjection with reference standard (Additional file 1: Fig. S1 and S2) confirmed the tracer identity and were used to calculate the molar activity. Table 1 summarizes the QC tests carried out and their results.

**Table 1:** Quality control (QC) results of [^11^C]3MeO4AP productions (n = 4)

Test	Result	Acceptance criteria	Method
Appearance	Pass	Clear and colorless solution	Visual inspection
Radiochemical identity	Pass	The retention time difference < 10%	Coinjection of non-radioactive 3MeO4AP and [^11^C]3MeO4AP using radioHPLC
Amount of unlabeled compound per injected dose	0.9 ± 0.1 μg	≤ 10 μg	Dose calibrator and analytical HPLC
Radiochemical purity	99 ± 1%	> 95%	RadioHPLC
Radionuclidic identity	Pass	Half-life = 20.3 ± 1 min	Dose calibrator
	Pass	> 99% of emissions at 511 keV or 1.022 MeV or Compton scatter	Gamma spectroscopy
Filter integrity	Pass	> 50 psi	Bubble point test
Bacterial Endotoxins Test (BET)	Pass	< 5 EU/mL	Endo safe NexGen (Charles River)
Sterility testing^b^	*Not performed*	No growth in TSB or FTM media after 3–5 days	Aerobic and anaerobic cultures
Chemical impurities per injected dose	Pass	< 5 μg	Dose calibrator and analytical HPLC
Residual solvent analysis^c^	*Not performed*	EtOH < 10% V/V DMSO < 3.3 mg/mL	Gas chromatography
Tracer stability	Pass	< 5% decomposition 2 h post injection	RadioHPLC
pH	7 ± 0.5	5–8	pH test strips
Osmolarity^c^	*Not performed*	Isotonic (250–350 mOsm/kg)	Osmometer

^a^Very high molar activity is not required for this tracer and was not optimized. Acceptance criteria was selected as an arbitrary minimum value based on our experience with C-11 labeled tracers

^b^Sterility testing was not required for non-human studies

^c^Residual solvent analysis and osmolarity testing were not required for non-human studies

### Biodistribution and radiation dosimetry of [^11^C]3MeO4AP

Two monkeys (one male, one female) were used in the study in order to be able to assess the dosimetry to reproductive organs. The subject characteristics are shown in Table 2.

**Table 2:** Summary of subject information

Rhesus macaques	Sex	Weight (kg)	Height (m)	Injected activity	Injected mass (μg)	Effective dose (ICRP 103) (mSv)
M1	Male	13.68	1.2	293.4 MBq (7.93 mCi)	0.700	1.05
M2	Female	9.74	0.91	290.5 MBq (7.85 mCi)	0.693	1.27

**Fig. 2:**
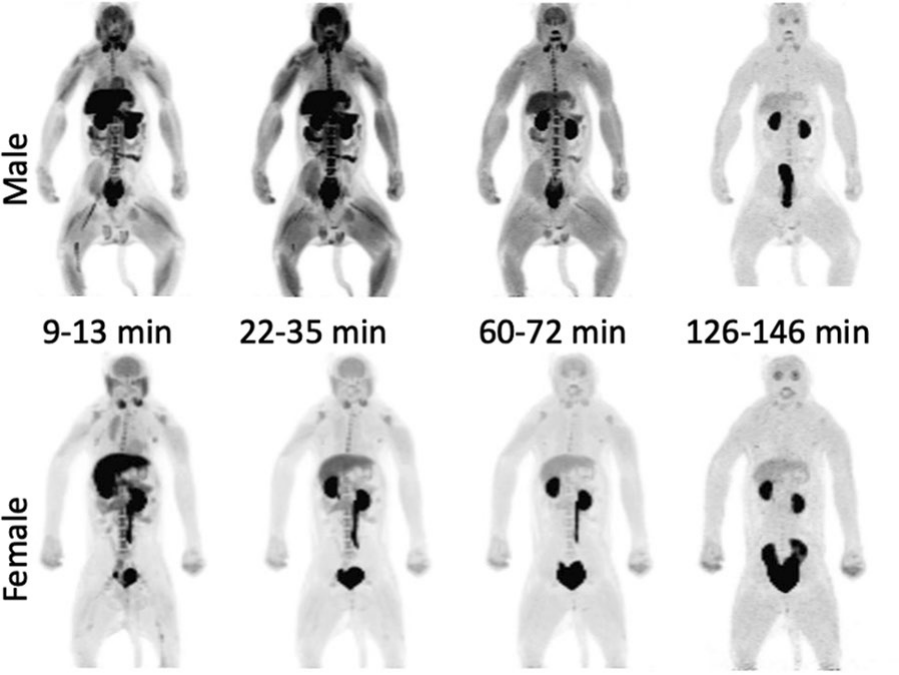
Whole-body maximum intensity projection (MIP) PET images (9–13 min, 22–35 min, 60–72 min, and 126–146 min) of rhesus macaques (male: top, female: bottom)

**Fig. 3:**
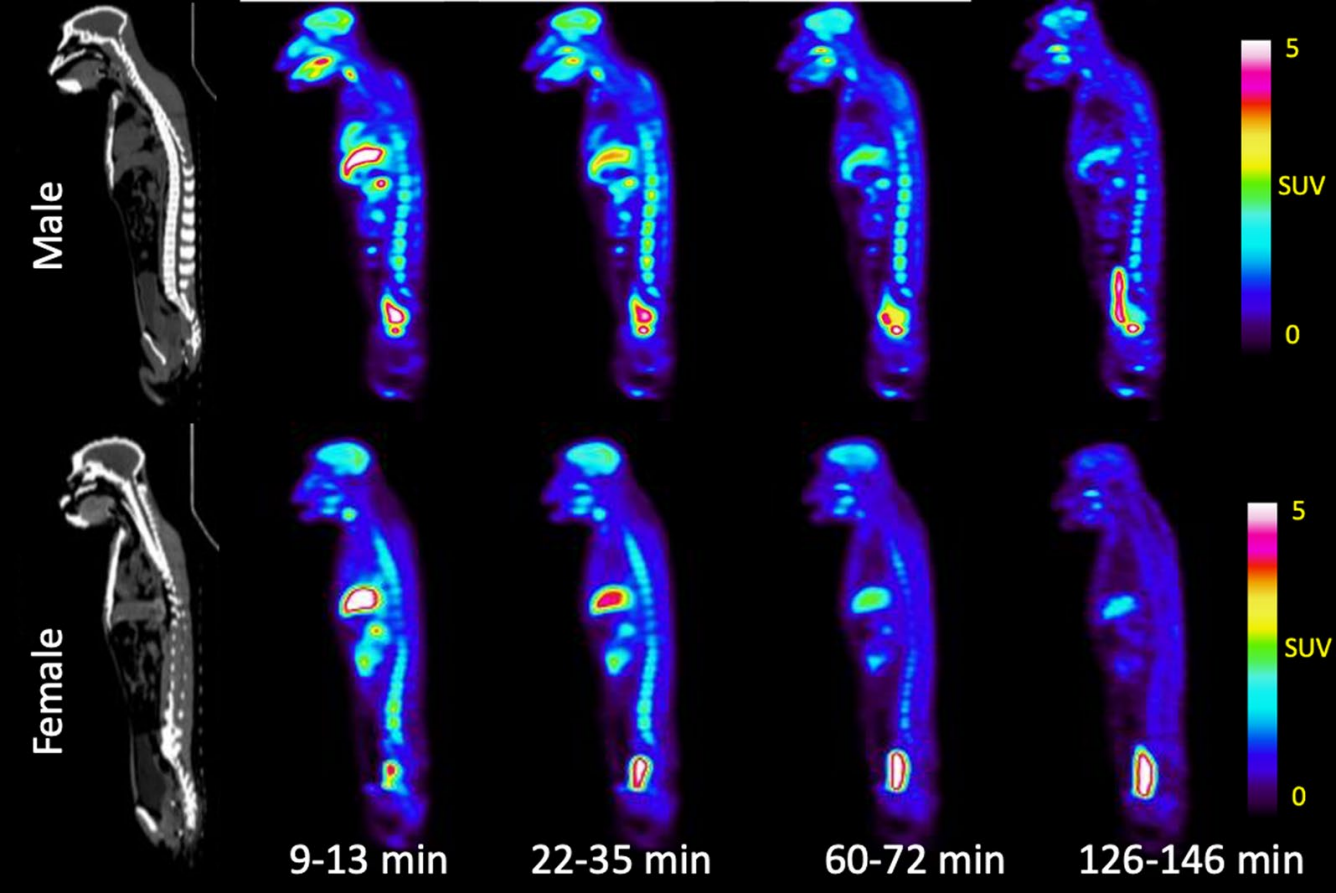
Representative whole-body CT and PET images (9–13 min, 22–35 min, 60–72 min, and 126–146 min) of rhesus macaques (Sagittal view, male at top, female at bottom)

**Fig. 4:**
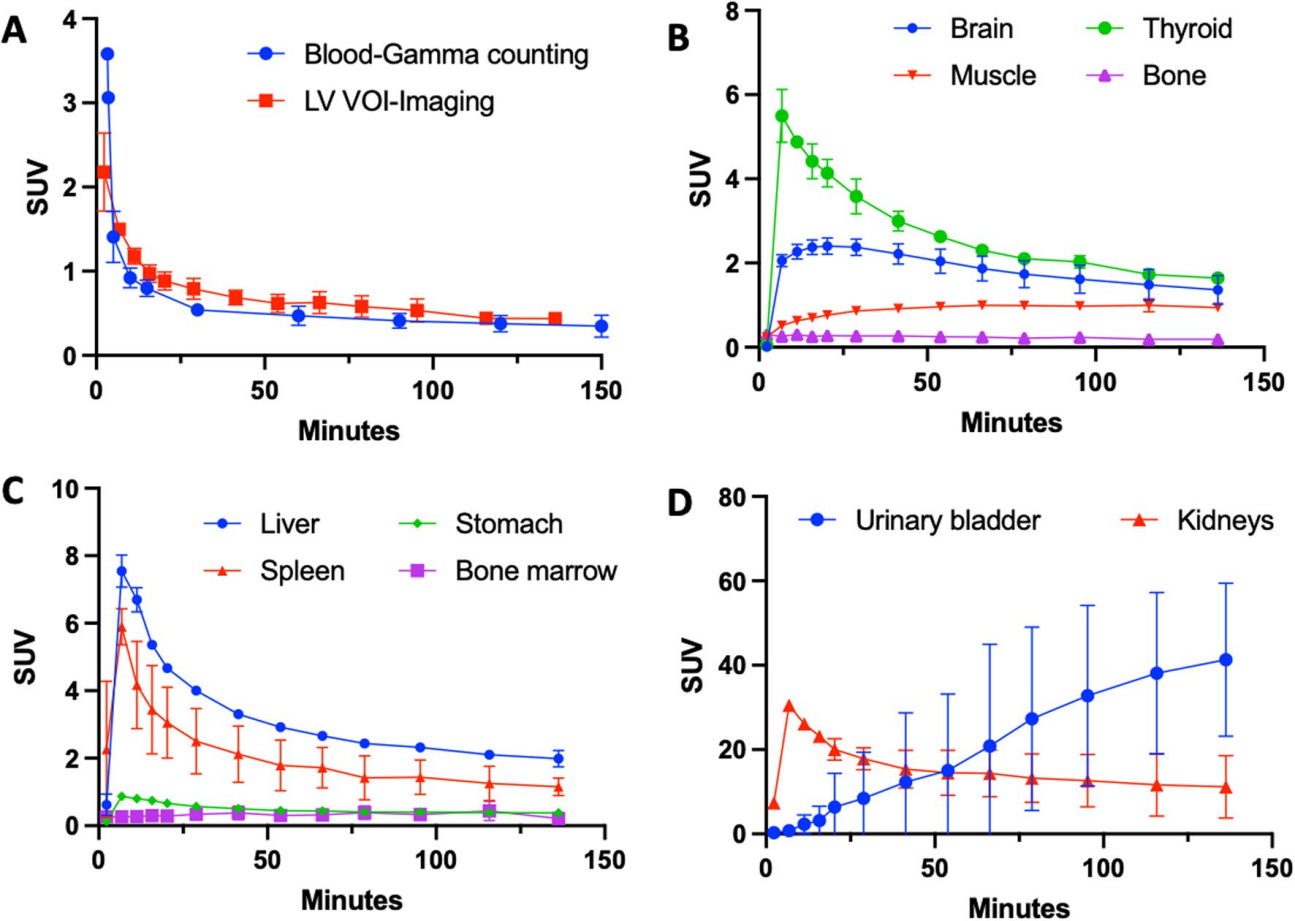
Organ specific time activity curves. **A** Whole blood decay-corrected time-activity curves measured by gamma counting and left ventricle (LV) VOI of PET imaging. **B–D** Decay corrected time-activity curves of selected organs (mean ± S.D. of 2 monkeys). Dots represent the mean value and bars the range for the two animals

*Whole body biodistribution*: As it can be seen from Fig. 2 (maximum intensity projections) and Fig. 3 (whole body sagittal slices) [^11^C]3MeO4AP quickly distributed widely throughout the body. Accumulation of tracer is clearly visible in the urinary bladder, kidneys, liver, thyroid, brain, salivary glands, and spinal vertebras. Figure 4A shows the TACs of blood obtained by gamma counting of serial arterial blood samples as well as a VOI placed in the heart left ventricular chamber. In addition, TACs were extracted from VOIs placed in brain, thyroid, muscle, and bone (Fig. 4B); liver, stomach, spleen, and bone marrow (Fig. 4C) and kidneys and bladder (Fig. 4D).

*Blood kinetics*: The concentration in blood peaked at 0–3 min post injection and then it quickly decreased. The blood radioactivity clearance was fitted using a two-phase decay model indicating fast washout (male: t_1/2 fast_ = 0.68 min, t_1/2 slow_ = 9.3 min; female: t_1/2 fast_ = 0.50 min, t_1/2 slow_ = 19.1 min). Comparison of the blood measured by gamma counting and from a VOI placed in the hearth left ventricle showed similar results highlighting the accuracy of both methods. From the images and TACs, it appears that the tracer is primarily cleared through the kidneys with the signal in the urinary bladder surpassing that of the kidneys after ~ 55 min (Fig. 4B). In most organs (e.g. kidneys, liver, spleen, stomach, thyroid, etc.) maximum SUV was reached during at 2–11 min post-injection. Meanwhile, the SUV of muscle increases until 20 min post injection and remains stable at SUV ≈ 1 until the end of imaging.

**Fig. 5:**
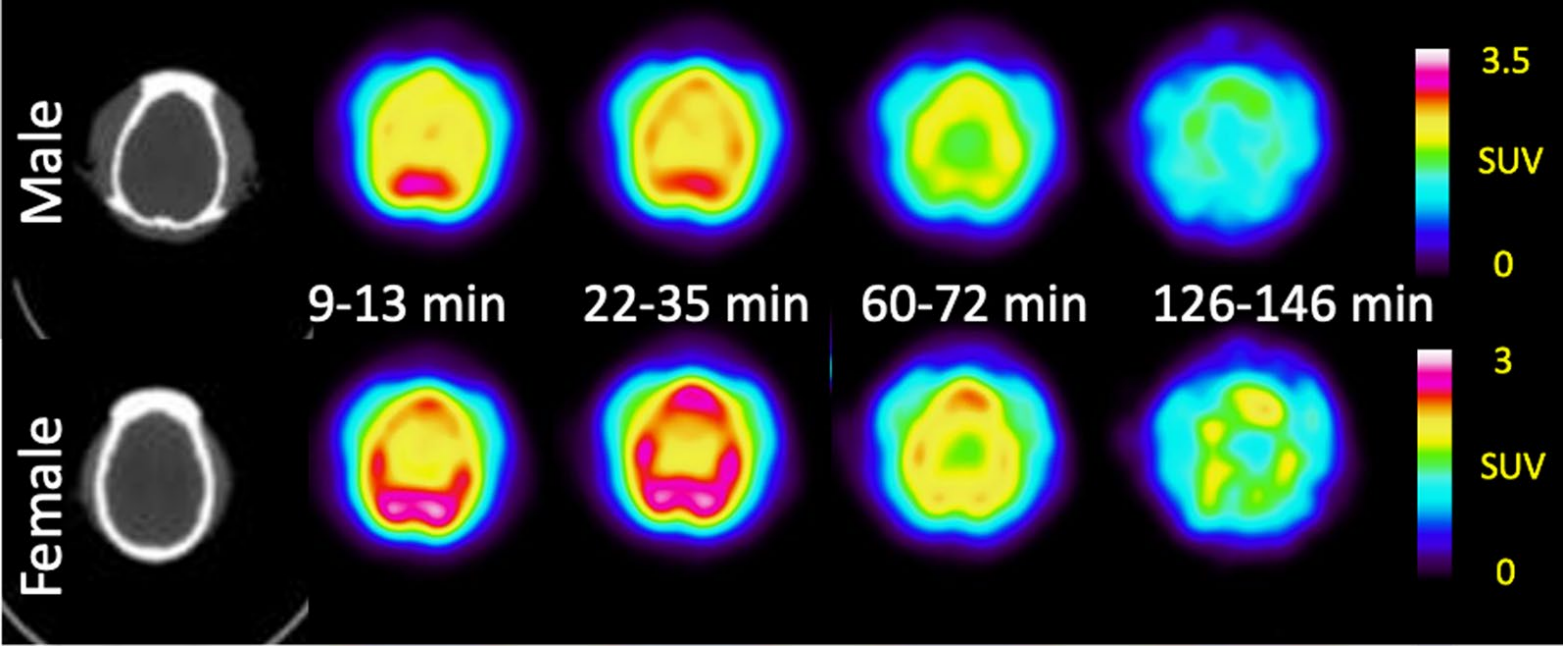
Representative brain CT and PET images (9–13 min, 22–35 min, 60–72 min, and 126–146 min) of rhesus macaques (male-top, female-bottom)

*Brian kinetics*: Consistent with previous results [10], the pharmacokinetics in the brain were slower than other organs, reaching a whole brain SUV ≈ 2.4 at 16–29 min post-injection followed by a slow decrease to SUV ≈ 1.4 by the end of imaging (150 min post injection). The dynamic changes in the brain are also apparent in horizontal brain slices (Fig. 5), which show higher signal in grey matter than white matter as previously described [10]. There were no significant differences observed between male and female.

*Residence time*: From the integration of the non-decay corrected TACs, the residence time of selected organs were calculated (Table 3). The brain, heart content (blood), kidneys, liver, and muscle of both male and female animal show relative longer residence time ranging from ~ 0.02 MBq*h/MBq to ~ 0.12 MBq*h/MBq. Meanwhile, the gallbladder contents, large intestines, testes, and thymus show relatively shorter residence time ranging from ~ 0.00011 MBq*h/MBq to ~ 0.00051 MBq*h/MBq.

**Table 3:** Residence times of [^11^C]3MeO4AP for measured organs and remainder of body

Organ	Residence time (MBq*h/MBq)	
	Male	Female
Adrenals	4.73 × 10^–04^	6.09 × 10^–04^
Brain	2.02 × 10^–02^	1.92 × 10^–02^
Breasts	1.33 × 10^–03^	2.01 × 10^–03^
Gallbladder Contents	2.29 × 10^–04^	2.26 × 10^–04^
LLI	6.84 × 10^–04^	3.11 × 10^–04^
Small Intestine	6.61 × 10^–03^	8.17 × 10^–03^
Stomach	6.90 × 10^–04^	6.18 × 10^–04^
ULI	6.84 × 10^–04^	3.11 × 10^–04^
Heart Contents	4.22 × 10^–02^	3.37 × 10^–02^
Heart Wall	3.53 × 10^–03^	2.94 × 10^–03^
Kidneys	4.17 × 10^–02^	4.04 × 10^–02^
Liver	5.36 × 10^–02^	5.25 × 10^–02^
Lungs	8.58 × 10^–03^	4.40 × 10^–03^
Muscle	1.48 × 10^–01^	1.06 × 10^–01^
Ovaries	NA	8.69 × 10^–05^
Pancreas	1.39 × 10^–03^	1.81 × 10^–03^
Red Marrow	1.49 × 10^–03^	4.65 × 10^–03^
Cortical Bone	7.13 × 10^–03^	1.30 × 10^–02^
Trabecular Bone	1.87 × 10^–03^	1.89 × 10^–03^
Spleen	4.20 × 10^–03^	3.24 × 10^–03^
Testes	3.59 × 10^–04^	NA
Thymus	9.02 × 10^–05^	1.26 × 10^–04^
Thyroid	4.69 × 10^–04^	5.14 × 10^–04^
Urinary bladder contents	8.91 × 10^–04^	5.19 × 10^–03^
Uterus/uterine wall	NA	1.61 × 10^–03^
Remainder	8.64 × 10^–02^	1.16 × 10^–01^

*Radiation dosimetry*: From the residence times, organ dosimetry data was calculated using ICRP 60 and ICRP 103 weighting factors (Table 4). The organs with the greatest contribution to the effective dose were the kidneys, liver, lungs, and testes. The calculated effective dose was 4.3 ± 0.6 μSv/MBq according to ICRP 60 or 4.0 ± 0.6 according to ICRP 103. The male animal effective dose was consistent with the female data. The effective dose of carbon-11 tracer [^11^C]3MeO4AP is notably lower than the fluorine-18 analogue [^18^F]3F4AP (measured in human: 12.2 ± 2.2 μSv/MBq [20]; measured in NHPs: 21.6 ± 0.6 μSv/MBq [12]) but comparable to the effective dose of other carbon-11 tracers measured in humans (*e.g*. [^11^C]choline: 4.4 μSv/MBq [21], [^11^C]glucose: 4.3 μSv/MBq [22]).

**Table 4:** Organ radiation dosimetry calculations of [^11^C]3MeO4AP using OLINDA software. Mean and range of values (n = 2)

Target organ	ED contribution ICPR 60 (μSv/MBq)		ED contribution ICPR 103 (μSv/MBq)	
Adrenals	0.028 ± 0.006		0.11 ± 0.03	
Brain	0.012 ± 0.001		0.051 ± 0.004	
Breasts	0.11 ± 0.02		0.26 ± 0.05	
Gallbladder wall	0		0.035 ± 0.004	
LLI wall	0.25 ± 0.02		0.25 ± 0.02	
Small intestine	0.010 ± 0.002		0.039 ± 0.008	
Stomach wall	0.30 ± 0.04		0.30 ± 0.04	
ULI wall	0.006 ± 0.001		0.30 ± 0.04	
Heart wall	0		0.158 ± 0.006	
Kidneys	0.98 ± 0.04		0.362 ± 0.014	
Liver	0.58 ± 0.10		0.46 ± 0.08	
Lungs	0.42 ± 0.04		0.42 ± 0.04	
Muscle	0.006 ± 0.006		0.023 ± 0.003	
Ovaries	0.70		0.28	
Pancreas	0.018 ± 0.003		0.066 ± 0.013	
Red marrow	0.25 ± 0.05		0.25 ± 0.05	
Osteogenic cells	0.031 ± 0.011		0.032 ± 0.012	
Skin	0.011 ± 0.002		0.011 ± 0.002	
Spleen	0.020 ± 0.001		0.073 ± 0.001	
Testes	0.60		0.24	
Thymus	0.008 ± 0.001		0.029 ± 0.003	
Thyroid	0.36 ± 0.06		0.29 ± 0.05	
Urinary bladder wall	0.20 ± 0.15		0.16 ± 0.12	
Uterus	0.010 ± 0.009		0.04 ± 0.03	
Effective dose (μSv/MBq)	Male	Female	Male	Female
	3.9	4.7	3.6	4.4
Mean effective dose (μSv/MBq)	4.3 ± 0.6		4.0 ± 0.6	

### Safety assessment

No changes in vital signs (including temperature, blood pressure and oxygen saturation, heart rate, respiratory rate, and exhaled CO_2_) were observed during the 150 min of PET imaging acquisition. Routine observation in housing during subsequent days revealed no indications of delayed adverse reaction.

## Discussion

The radiosynthesis of [^11^C]3MeO4AP was achieved using the GE TRACERlabT FX C Pro synthesizer. The previously reported semi-automated radiosynthesis method required the manual addition of reagent into the reaction vessel and manually loading the reaction mixture into the prep-HPLC. The operator was exposed to low amounts of radiation during this process and such manual operation does not fulfill the cGMP requirement. The newly developed fully automated radiosynthesis process achieved fully remote-controlled synthesis and fulfills cGMP requirements. The final dose passed all the required QC tests. The molar activity was moderate but high molar activity is not critical for imaging with this tracer, since our previous studies have shown that addition of cold tracer does not prevent binding to lesions [10, 12]. The total synthesis and purification time of fully automated method is *ca*. 5 min shorter than the semi-automated method. Even though, the non-decay corrected radiochemical yield of the automated method is 3% lower, the yield is sufficient to produce doses for non-human primate use and can easily be scaled-up to produce human doses in the future.

The biodistribution study shows widespread distribution and fast clearance from most organs. The liver, spleen and thyroid show high initial SUV and slow washout likely due to high blood perfusion and binding of the tracer to voltage-gated potassium channels in these organs [7]. The high SUVs of kidneys and urinary bladder confirms that the [^11^C]3MeO4AP undergoes primarily renal clearance and is eventually eliminated in the urine. The whole body biodistribution of [^11^C]3MeO4AP is similar to that of [^18^F]3F4AP, except that there is lower uptake in the stomach. The slower kinetics in the brain compared to other organs are consistent with previous reports and suggest a high level of specific binding in the brain compared to other organs. The increasing muscle SUVs are most likely due to binding since there is high expression of voltage-gated potassium channels in muscle [7]. There were no differences between male and female data. Despite the small number of subjects, the data exhibits a high degree of consistency, indicating its reliability.

[^11^C]3MeO4AP shows low radiation dosimetry based on OLINDA calculation. The effective dose is ~ 35% of [^18^F]3F4AP (12.2 ± 2.2 μSv/MBq [20], human data) and similar to [^11^C]choline (4.4 μSv/MBq [21], human data), and [^11^C]glucose (4.3 μSv/MBq [22], human data). A patient receiving ~ 400 MBq of [^11^C]3MeO4AP will receive ~ 1.7 mSv of radiation dose from the PET tracer, which is well within the limits typically allowed for human research studies.

### Supplementary Information

**Additional file 1:** Supplementary Information.

## Data Availability

The datasets generated and analyzed in this study are available from the corresponding authors upon request.
