# Supplementary material for: Radiosynthesis automation, non-human primate biodistribution and dosimetry of K+ channel tracer [11C]3MeO4AP
Source: EJNMMI Res. 2024 Apr 29;14:43. doi: 10.1186/s13550-024-01092-8 (PMC11058135; doi:10.1186/s13550-024-01092-8)
Supplement: Supplementary file 1 — Additional file 1: Supplementary Information. [file 13550_2024_1092_MOESM1_ESM.docx]

**Supporting Information**

Radiosynthesis automation, non-human primate biodistribution and dosimetry of K^+^ channel tracer [^11^C]3MeO4AP

**Authors:**

Yu-Peng Zhou^1^, Moses Q. Wilks^1^, Maeva Dhaynaut, Nicolas J. Guehl, Danielle R. Vesper, Sung-Hyun Moon, Peter A. Rice, Georges El Fakhri, Marc D. Normandin* and Pedro Brugarolas*

**Affiliations:**

Gordon Center for Medical Imaging, Massachusetts General Hospital and Harvard Medical School, Boston, Massachusetts, United States.

^1^ Yu-Peng Zhou and Moses Q. Wilks contributed equally

***Correspondence:**

Pedro Brugarolas, PhD

55 Fruit St

Bulfinch 051

Boston, MA 02114, United States.

[pbrugarolas@mgh.harvard.edu](mailto:pbrugarolas@mgh.harvard.edu)

Marc D. Normandin, PhD

55 Fruit St

White 427

Boston, MA 02114, United States.

[normandin@mgh.harvard.edu](mailto:normandin@mgh.harvard.edu)


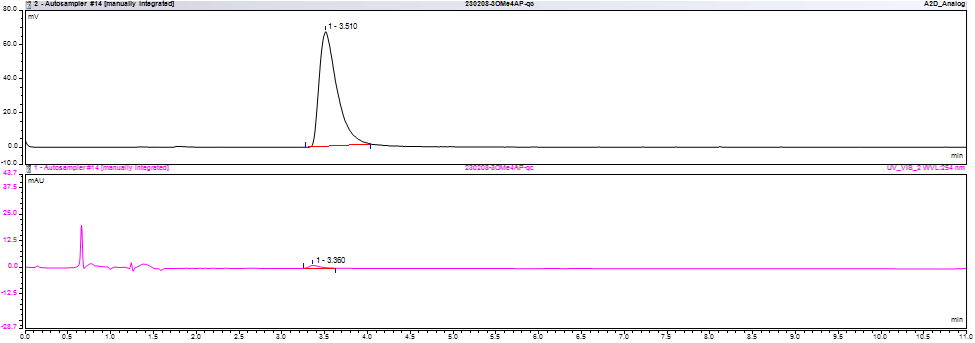


Figure S1. HPLC chromatogram of [^11^C]3MeO4AP dose (top: radiodetector; bottom: UV detector at 254 nm)


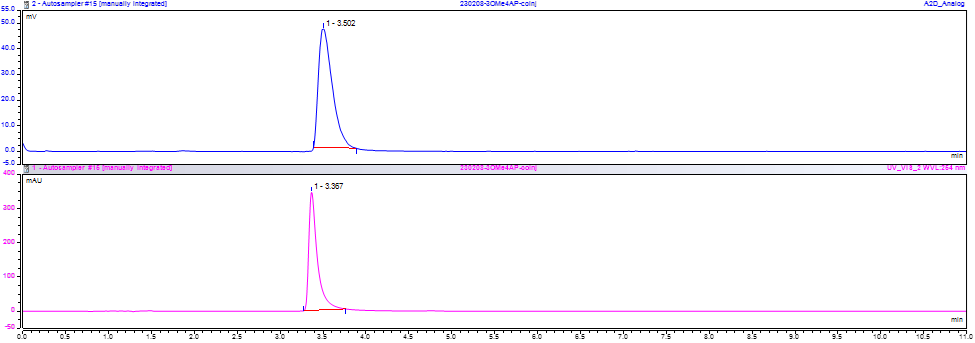


Figure S2. HPLC chromatogram of [^11^C]3MeO4AP dose with co-injection of non-radioactive 3MeO4AP (top: radiodetector; bottom: UV detector at 254 nm)
